# Molecularly Targeted Agents as Radiosensitizers in Cancer Therapy—Focus on Prostate Cancer

**DOI:** 10.3390/ijms140714800

**Published:** 2013-07-16

**Authors:** Sara Alcorn, Amanda J. Walker, Nishant Gandhi, Amol Narang, Aaron T. Wild, Russell K. Hales, Joseph M. Herman, Danny Y. Song, Theodore L. DeWeese, Emmanuel S. Antonarakis, Phuoc T. Tran

**Affiliations:** 1 Department of Radiation Oncology and Molecular Radiation Sciences, Sidney Kimmel Comprehensive Cancer Center, Johns Hopkins University School of Medicine, Baltimore, MD 21231, USA; E-Mails: salcorn2@jhmi.edu (S.A.); awalke46@jhmi.edu (A.J.W.); nishantngandhi@gmail.com (N.G.); anarang2@jhmi.edu (A.N.); awild1@jhmi.edu (A.T.W.); rhales1@jhmi.edu (R.K.H.); jherma15@jhmi.edu (J.M.H.); dsong2@jhmi.edu (D.Y.S.); deweese@jhmi.edu (T.L.D.); 2 Department of Oncology, Sidney Kimmel Comprehensive Cancer Center, Johns Hopkins University School of Medicine, Baltimore, MD 21231, USA; E-Mail: eantona1@jhmi.edu; 3 Department of Urology, Johns Hopkins University School of Medicine, Baltimore, MD 21231, USA

**Keywords:** prostate cancer, radiotherapy, radiosensitizer, molecularly targeted agents, HSP90 inhibitors

## Abstract

As our understanding of the molecular pathways driving tumorigenesis improves and more druggable targets are identified, we have witnessed a concomitant increase in the development and production of novel molecularly targeted agents. Radiotherapy is commonly used in the treatment of various malignancies with a prominent role in the care of prostate cancer patients, and efforts to improve the therapeutic ratio of radiation by technologic and pharmacologic means have led to important advances in cancer care. One promising approach is to combine molecularly targeted systemic agents with radiotherapy to improve tumor response rates and likelihood of durable control. This review first explores the limitations of preclinical studies as well as barriers to successful implementation of clinical trials with radiosensitizers. Special considerations related to and recommendations for the design of preclinical studies and clinical trials involving molecularly targeted agents combined with radiotherapy are provided. We then apply these concepts by reviewing a representative set of targeted therapies that show promise as radiosensitizers in the treatment of prostate cancer.

## 1. Introduction to Targeted Radiosensitizers

Radiotherapy (RT) is a mainstay of cancer treatment, offering both definitive and palliative strategies for disease management. An evidence-based estimate of the proportion of patients with cancer for whom external beam RT is indicated is 52% [1]. RT plays a pivotal role in attaining cure rates in a variety of cancer types; a report from the Royal College of Radiologists estimates that based on its relative contribution, RT is responsible for 40% of cures, whereas chemotherapy contributes to 11% and surgery to 49% of cures [2]. Additionally, RT has a multitude of palliative uses—For example providing significant symptom improvement for 50%–80% of cases of painful bone metastases [3].

While RT provides local control of the primary tumor, the addition of systemic treatment can potentially manage occult distant disease and afford additional radiosensitization benefits [4]. The combination of cytotoxic chemotherapy and RT has become increasingly common over the past 30 years, likely in part due to the increased use of RT in place of primary surgery and the trend toward inclusion of neoadjuvant and adjuvant chemotherapy regimens [5]. Yet the addition of conventional cytotoxic chemotherapy for radiosensitization often comes at the cost of increased toxicity due to lack of specificity for tumor cells. Thus to further improve the effects of RT while minimizing normal tissue sensitization, an area of evolving interest is the application of agents that selectively target tumor-specific pathways thought to be important in radiation-induced cell death. In contrast with conventional cytotoxic chemotherapies, many of the molecularly targeted agents currently studied for use as radiosensitizers are cytostatic [6], exploiting differences between malignant and nonmalignant cells with relative sparing of normal tissues.

Agents expected to modify tumor response to RT generally do so by altering one or more of the “5 Rs of radiobiology”: Inherent cellular radiosensitivity, repair, reassortment, repopulation, and reoxygenation [7]. Indeed, two of the best supported targeted radiosensitizers can be classified in this manner: Nimorazole and cetuximab. Nimorazole functions as a selective tumor radiosensitizer by preferentially targeting hypoxic tumor cells, which are otherwise relatively resistant to radiation. In combination with definitive RT for patients with cancer of the supraglottic larynx and pharynx in a phase III trial, the addition of nimorazole significantly improved locoregional control by 16% without excess toxicity as compared to RT alone [8]. However, this agent failed to become adopted as the standard of care given multiple factors as reviewed by Overgaard *et al.* [9], and is only routinely used in Denmark [6]. Cetuximab is an epidermal growth factor receptor (EGFR) antagonist that decreases cellular proliferation and promotes apoptosis by hampering the pro-survival pathways fostered by EGFR overexpression in tumor cells. In a phase III trial, the addition of cetuximab to definitive RT in patients with squamous head and neck cancers improved 5-year overall survival by 10%, while the side effect profile between placebo and cetuximab treatment arms was comparable [10]. Such molecularly targeted agents—Shown to improve tumor control without resulting in untoward normal tissue toxicity—Demonstrate the considerable potential for the development of molecularly targeted radiosensitizers to be used in combination with RT.

Despite the substantial possible benefit of combining novel targeted agents with RT, relatively few such combinations have been used in clinical trials. As such, there are even fewer examples of successful implementation of such agents into routine clinical practice, as highlighted by the rare examples of nimorazole and cetuximab above. Whereas there are an estimated 400 phase I non-RT oncology trials per year [11], there were only approximately 30 RT-related phase I and I/II trials in 2009 [12]. This may be due in part to several limitations specific to combined radiosensitizer and RT trials, including funding and trial development considerations, difficulties in identification of the optimal patient population, and additional pre-clinical and phase I trial-specific limitations. Due to a lack of formal guidelines for early stage preclinical and clinical development of radiosensitizers, cooperative groups including the RTOG and the NCI as well as at least one pharmaceutical company have recently published guidelines and strategies for performing preclinical and clinical studies with radiosensitizers. Herein we provide a brief summary of these recommendations [6,11,13–15].

## 2. Recommendations for Preclinical Studies with Radiosensitizers

*In vitro* and *in vivo* studies are a starting point for discovery of novel molecularly targeted radiosensitizing agents. Through biomarker discovery and establishing proof-of-concept principals, preclinical studies also lay the framework for incorporation of translational endpoints into trial design. Perhaps most importantly, preclinical studies are necessary prior to moving forward with large-scale clinical trials where patients may be exposed to potentially toxic therapy. Despite limited preclinical models for normal tissue toxicity, *in vivo* studies should demonstrate that treatment-related normal tissue toxicities are not beyond what is reasonably expected.

### 2.1. Overview of Limitations

Despite the importance of preclinical studies, there are several limitations and barriers specific to preclinical development of novel radiosensitizers. First, there is little consensus on what pre-clinical data is needed to support progression into the clinical trial phase [6]. Furthermore, novel radiosensitizer trials are uncommon and require the formation of typically uncharted relationships between players such as experts in the pharmaceutical industry and radiobiology. From the perspective of pharmaceutical companies, there may be a financial disincentive to perform thorough preclinical and early phase trial investigations of the interactions of such combination therapies, as this may prolong the time a particular agent is in pre-clinical development [11]. An additional RT-specific pre-clinical limitation is the applicability of animal models in this setting, due to tumor- and size-specific considerations. Tumors in animal models are relatively small and are often irradiated over the course of days whereas the corresponding tumor in humans may be relatively large and require RT over protracted weekly treatment courses [6]. These variables may contribute to relative radioresistance in humans, and must be accounted for when applying pre-clinical data derived from such animal models towards the design of clinical trials. The rapid development of molecular targeted agents over the past 10–15 years has created an opportunity for the study of these agents as radiosensitizers. As a result, recommendations and guidelines have been published to address these limitations, which we will summarize below [11,14].

### 2.2. Rational Target Selection

Given the number of candidate targeted agents that are in the early phases of development, it is nearly impossible to test every combination with radiation. However, many novel agents have mechanisms of action that are well-positioned to serve as radiation enhancers—Some of which have a promising role in prostate cancer and will be described later in this review. A possible first step for general target identification is to compile a catalog of molecular pathways that are known to have an important role in the biological behavior of cancers, specifically focusing on pathways considered to be integral in modulating response to irradiation. The next step is to cross-reference this list of pathways or molecules that are actionable (“druggable”) with targeted agents that are already in preclinical and clinical development [11].

Another approach proposed by Lin *et al.* is to again start with targeted agents that are in later phases of preclinical and clinical development but then focus research efforts only on those agents with established biomarkers predictive of clinical benefit, such as mutations or target protein overexpression [14]. For such agents, evidence of a clear on-target effect has usually been established. An example is erlotinib, an EGFR tyrosine kinase inhibitor (TKI) that has demonstrated clinical benefit in tumors that harbor activating EGFR mutations. Preclinical testing should focus on confirming activity when combined with radiation and identifying mechanisms of resistance.

### 2.3. *In Vitro* Studies

Although *in vitro* studies often do not address all the complexities and nuances of cancer biology, they are a necessary starting point in preclinical development. *In vitro* studies typically include cell lines in standard tissue culture or in three-dimensional culture and are conducted to demonstrate agent activity, target knockdown and potential tumor selectivity, as well as to elucidate mechanism of action and resistance pathways. Molecularly targeted agents can be broadly grouped into tumor-specific and tumor non-specific groups. For those agents that are hypothesized to interact with targets that are aberrantly expressed in a wide range of cancers, the selection of cell lines should be based on knowledge of expression of the target with consideration of what types of tumors will be studied in clinical trials [6,11,14]. For targeted agents with a more limited scope, it is appropriate to focus on cell lines (preferably at least two) that overexpress the target of interest [11]. Experiments should be designed to allow derivation of dose enhancement ratio (DER) or sensitizer enhancement ratios (SER) [16,17]. DER is equal to the surviving fraction at an indicated radiation dose divided by the surviving fraction at the same dose of radiation plus the potential sensitizer. Measurement of cell death with clonogenic survival assay is the gold standard and is certainly necessary when performing SER and DER experiments [18]. In rare situations, colorimetric viability assays may be reasonable substitutes [11].

### 2.4. Statistical Analysis of Combination Studies

The aims of combining a systemic agent with RT are to achieve a synergistic (or additive) therapeutic effect with acceptable toxicity and to minimize or delay the induction of radiation- and/or drug-resistance. In order to avoid common errors and pitfalls in combination studies, there has been a push from the scientific community to create a standard definition for synergy [19]. One popular and widely accepted method was jointly introduced by Chou and Talalay in the 1980s. They introduced the term “combination index” (*CI*) to quantitatively depict synergy (*CI* < 1), additive effect (*CI* = 1), and antagonism (*CI* > 1) [20]. This approach views synergy as a reaction, operating on physiochemical mass-action laws rather than a statistical consideration. Therefore, they proposed that one should determine synergy with *CI* values, not *p* values. The *CI* provides a quantitative measure of the degree of interaction between two or more agents. A slight modification of this method was optimized for combination studies with RT where experiments are performed using a range of doses in a non-constant ratio checkerboard design in order to derive a *CI* [21].

### 2.5. *In Vivo* Studies

*In vivo* studies are particularly important when examining agents that act on the tumor microenvironment such as anti-angiogenic agents. Before therapeutic efficacy studies are performed with animal models (typically murine), it is ideal to have demonstrated a suitable pharmacokinetic profile of the drug in the mouse. Furthermore, it is important that the drug reach active concentration levels in animal tumors and that downstream modulation of the target can be measured. The majority of *in vivo* studies involve immunocompromised mice including athymic, severe combined immune-deficiency (SCID) or NOD-SCID mice that have mutations in both DNA response and repair pathways. It is not surprising, however, that some of the anti-tumor effects of RT may be mediated by the immune system. Therefore, immunocompromised mice are not optimal in this regard given that they lack a fully functional immune system [11]. For this, among other reasons, genetically engineered mouse models (GEMMs) are becoming more popular for preclinical studies with and without RT. One example of this is an inducible triple transgenic autochthonous mouse model of lung adenocarcinoma (*CCSP-rtTA/tetO-Kras^G12D^/Twist1-tetO_7_-luc*), which our group has used recently to show the radiosensitizing properties of Hedgehog pathway inhibition on the tumor microenvironment [22]. Recently, the Mouse Models of Human Cancer Consortium have published a consensus statement of useful preclinical prostate cancer mouse models [23].

More sophisticated animal studies with RT are now possible with the advent of technologies that integrate imaging, treatment planning, and radiation delivery capabilities such as the small-animal radiation platform (SARRP) and the microRT small animal conformal irradiator [24,25]. In terms of dose and fractionation, abbreviated fractionations are reasonable for proof-of-principal studies, especially given that there is a recent trend towards hypofractionated regimens in the clinic with the advent of stereotactic body radiotherapy (SBRT)/stereotactic ablative body radiation (SABR) and more conformal therapy. While more conventional fractionated courses of RT may be more appropriate for studying toxicity [11], normal tissue toxicity models and protracted dosing studies where daily RT doses of 2 Gy are given with repeat doses of radiosensitizer are often expensive, time consuming, and cumbersome.

Normal tissue toxicity is difficult to assess *in vitro* and *in vivo*. While there are some useful preclinical models such as the intestinal crypt colony assay or the ventral tongue mucosal assay, these are limited to a few institutions [26,27]. Surrogate endpoints include *in vitro* analysis of the kinetics of DNA repair in normal tissues. A key “go/no-go” step in radiosensitizer development proposed by Harrington *et al.* is the evaluation of the relative degree of sensitization (as measured by SER) of tumor *vs.* normal tissues. Agents with a normal tissue SER that is greater than tumor SER should generally not proceed to clinical development. In certain situations where the target is known and the preclinical models are not optimal, it is reasonable to proceed directly to the clinic without testing for normal tissue toxicity. This is particularly true for agents that have completed pharmacokinetic and toxicity profiling in early stage clinical trials [11].

In summary, preclinical studies of radiation response modifiers have multiple purposes including demonstration of efficacy, exploration of mechanisms of action, identification of a target, investigation of optimal dose/fractionation of both the radiation and the systemic agent, and evaluation of normal tissue response. Despite the importance of preclinical studies, the inadequacy of our current models hampers the drug development process. There is a great need to improve upon our preclinical assays and animal models as they can provide valuable insight and expedite the development and implementation of successful human clinical trials involving radiosensitizers.

## 3. Recommendations for Clinical Trials with Radiosensitizers

### 3.1. Identification of Patient Populations

In trial design for novel radiosensitizers, selection of the optimal patient population is particularly difficult but crucially important. On one hand, while trials targeting patients with metastatic and refractory cancer may allow for assessment of toxicity, these trials are unlikely to afford high response rates or meet cost-benefit analysis thresholds for approval by regulatory agencies [11]. Yet conducting novel radiosensitizer trials in patients with curable disease raises ethical considerations, especially where there is an accepted standard-of-care. This is particularly true if toxicity from the radiosensitizer leads to delay or interruption of curative RT, which may reduce the probability of tumor control [11,28]. Identifying tumor types most amenable for clinical investigation requires further deliberation. To overcome the above ethical issues, solutions include studying cancers with poor prognosis but for which definitive management may still be attempted, such as pancreatic cancer, locally advanced lung cancer, or glioblastoma multiforme [11]. Other potentially useful tumor types include high-risk, locally-advanced prostate cancer, which allows for expedited evaluation through the use of intermediate endpoints such as progression-free survival or post-treatment biopsies as well as bladder cancer, which can be easily for biomarker studies [13].

### 3.2. Molecular Biomarkers and Functional Imaging

During preclinical and early phase clinical trials, efforts should be made to explore molecular biomarkers and functional imaging, which may provide evidence of proof of principle, target inhibition, or early signs of antitumor activity. Such biomarkers may serve to bridge the inadequacies of traditional clinical factors, thus opening the door for personalized treatment approaches that allow for tailoring of treatment options to maximize therapeutic outcome. Investigation of prostate cancer prognostic and predictive tissue-based molecular biomarkers is a prime example of research that may ultimately direct definitive and salvage RT treatment decisions [29]. Furthermore, incorporation of pre- and mid-treatment biopsies into clinical trials may aid in determining predictors of response. However, this may be impractical and unsafe in certain situations.

In addition to molecular biomarkers, functional (or dynamic) and molecular imaging has become an increasingly important assessment tool, as such techniques can address the key issue of distinguishing aggressive from indolent tumor types and may aid in predicting response to therapy [30–33]. Positron emission tomography (PET), particularly with ^18^F-fluorodeoxyglucose (^18^F-FDG PET), has gained an important role in the clinical management of cancer patients, particularly for staging and assessment of response to therapy [34,35]. However, there are limitations of ^18^F-FDG PET. In prostate cancer, studies employing ^18^F-FDG PET have demonstrated low uptake except for advanced metastatic disease [36–39]. A number of novel PET radiotracers are being investigated for use in prostate cancer, but none have yet gained widespread clinical use [40,41]. ^11^C-choline is the most widely studied experimental PET radiotracer for detection of prostate cancer and has demonstrated utility in detection of lymph node and bone metastases [42–44]. ^11^C-acetate is another emerging radiotracer, which has been evaluated in a limited number of studies and demonstrates comparable uptake to ^11^C-choline for detection of primary and metastatic disease [45]. Sodium ^18^F-fluoride-PET (NaF-PET) has proven very sensitive for the identification of bone metastases but is unable to differentiate between viable metastatic prostate tumor and chronic reactive bone changes [46,47]. Other promising radiopharmaceuticals for prostate cancer include anti-1-amino-3-^18^F-fluorocyclobutane-1-carboxylic acid (^18^F-FACBC) and ^18^F-fluorodihydrotestosterone (^18^F-FDHT), which are also actively undergoing clinical evaluation in a variety of settings [48–50]. Further work is necessary to compare the merits of ^11^C-choline and other emerging PET radiotracers [51].

Prostate-specific membrane antigen (PSMA) is a well-characterized molecular biomarker for prostate cancer that has been associated with tumor aggressiveness. Histologic studies have associated high PSMA expression with metastasis [30,52,53] and androgen independence [31], and expression levels have been found to be predictive of prostate cancer progression [32,54]. More recently, in preclinical studies PSMA expression has been shown to correlate inversely with androgen receptor (AR) signaling, suggesting PSMA as a surrogate for AR signaling activity [55]. Previous attempts to image PSMA by single photon emission computed tomography (SPECT) (ProstaScint^®^) demonstrated poor performance due to the limitations of imaging with intact antibody (poor tumor penetration and slow blood pool clearance) and the inherently low resolution of SPECT [56,57]. Other lower molecular weight PET radiotracers targeting PSMA are being developed. For example (*N*-[*N*-[(*S*)-1,3-dicarboxypropyl]carbamoyl]-4-[^18^F]fluorobenzyl-l-cysteine) (DCFBC) and 2-(3-ureido)-pentanedioic acid (DCFPyL) [58]. As low-molecular-weight species, these compounds promise better pharmacokinetics than antibodies, and their radiosyntheses are highly amenable to automation and dissemination [58–60]. Clinical validation of this hypothesis is ongoing.

Current conventional imaging modalities in prostate cancer (computed tomography, bone scan, magnetic resonance imaging, ultrasound) have limited accuracy in the initial staging and for determining prognosis. A non-invasive, imaging-based biomarker to address the issue of aggressiveness, metastatic tumor burden, and degree of AR signaling activity would be a tremendous advance to improving risk stratification and therapeutic monitoring of cancer patients. Prospective validation of PSMA-based imaging as well as other molecular and functional imaging modalities is needed; therefore, it is strongly encouraged to incorporate such endpoints into clinical trial design. [14].

### 3.3. Toxicity

In terms of addressing the complex issue of toxicity in clinical trials with radiation modifiers, it has been generally accepted that phase I studies should focus on early effects. This is based on the assumption that severe late effects are associated with severe acute toxicity. However, this is not always the case, particularly in hypofractionated regimens where late effects may be severe in the absence of any short-term toxicity. To that end, it is appropriate to allow longer follow up in phase I and II trials before proceeding with phase III studies, and it is necessary to design phase II and III trials that allow collection of long-term toxicity data. Because there is often overlap between toxicity due to RT alone and toxicity from the systemic therapy, as well as potential additive toxicity when combining systemic agents with RT, an independent medical monitor knowledgeable about RT side effects should be assigned to each trial to adjudicate attributions of dose limiting toxicities (DLTs) [6]. Given that the toxicity from RT depends on the location of treatment (e.g., abdomen *vs.* brain) rather than the histologic type of tumor, it is recommended that palliative phase I trials be organ-specific, rather than solely disease specific [6,11].

### 3.4. Considerations in Trials with Curative Intent

An advantage of trials with curative intent is that the toxicities are inherently easier to compare between patients because treatment is often delivered to the same site (e.g., head and neck, lung, *etc*.). In addition, now more than ever, cost effectiveness is weighed heavily in regards to approval of novel agents [61]. As a result, there has been a push, particularly from the pharmaceutical companies towards exploring radiosensitizers in preclinical studies that could be used in definitive settings [13]. One important consideration in the design of trials with curative intent is the study endpoint. Despite long follow-up times necessary for overall survival benefit to be demonstrated in such clinical trials, more timely information could potentially be obtained for local control or progression-free survival (PFS). Furthermore, it is necessary to acknowledge that severe transient toxicity may be considered acceptable, as RT is a means to provide durable tumor control [6].

### 3.5. Considerations Specific to Phase I Clinical Trials

Designing phase I trials that combine RT with radiosensitizers can be challenging. It is important to note the differences between phase I trials with single-agent systemic therapies and phase I trials with radiosensitizers. For example, the extent to which normal tissues are exposed to RT depends on the site of treatment; this complicates estimation of toxicity and the decision for dose-escalation when different tumor sites are included in a trial involving RT. By comparison, side effects to organs at risk from systemic therapy alone are relatively independent of the tumor site and type, enabling trials to assess toxicity for a heterogeneous population of tumors [11]. It is important to note that Phase I studies with radiosensitizers should be designed to determine the dose of the systemic agent that is to be administered concurrently with a standard and appropriate dose and schedule of radiation, rather than administering fixed doses of systemic agent and titrating up the dose of RT. It should also be kept in mind that the recommended dose and biologically active dose may be different from the maximum tolerated dose, which is typically the goal in phase I studies with single-agent systemic therapies [62]. In some circumstances, defining a maximal dose based on toxicity may not be appropriate (e.g., for agents associated with very minimal toxicity or for agents for which escalation beyond a given dose may not be feasible because of absorption or financial constraints).

Toxicity assessment and dose escalation decisions are further hindered by the relatively high rate of grade 1–3 acute toxicities experienced with definitive RT. This complicates subsequent attribution of the side effect profile to the trial agent versus to the expected course of RT [11]. Moreover, the time for which toxicity is assessed must be longer for trials involving RT, where acute toxicity can occur even 8 to 12 weeks following treatment. In comparison, trials of cytotoxic drugs alone may achieve acute toxicity outcomes within days of administration [6]. The rate of dose escalation should be carefully considered and based upon expected toxicity and the degree of uncertainty involved. Another difference between phase I studies in single-agent systemic therapies and those that investigate radiosensitizers is that there is often not a need to incorporate pharmacokinetic studies into a radiation phase I trial, since this information has likely already been obtained from the single agent studies [6].

One of the most significant challenges in Phase I trial design with radiosensitizers is that the trials are prohibitively long because traditional trial designs, such as the classic 3 + 3 (cohort-of-three) design, require each patient or cohort of patients to be fully evaluated for the dose-limiting toxicity (DLT) before new patients can enroll [11]. Spinoffs of these trial designs aimed to reduce how often patient accrual is suspended and thereby shortening the duration include the Rolling Six Design (RSD) and the Continual Reassessment Method (CRM). The RSD allows for temporal overlap of the two cohorts of three subjects as long as at least one patient has been fully followed on that specified dose level [63,64]. Briefly, the CRM is a sequential sampling procedure that utilizes a mathematical model relating dose levels to the probability of DLT. The main advantage of the CRM is that it utilizes information from all previously treated patients continuously during the study to update estimates of DLT probability at each dose level. However, in the context of frequently encountered late toxicities that are important in radiation therapy trials, the CRM shares the same drawback with the 3 + 3 and RSD design in terms of prolonged trial duration. This is because the CRM involves treating one patient at a time and the dose assignment decision cannot be made for the next patient until DLT information is completely observed from prior patients [63,65].

A trial design that specifically addresses the late toxicity issues inherent in RT is the Time-to-Event Continual Reassessment Method (TITE-CRM), which allows staggered accrual without the need for complete DLT follow-up of previously treated patients [66,67]. However, in the setting of rapid patient accrual and late-onset toxicities, the TITE-CRM design may result in overly aggressive dose escalation and could expose a considerable number of patients to toxic doses of combination therapy. An alternative design has been proposed that is based on a two-stage approach that incorporates an accrual suspension rule to the TITE-CRM model according to a simple waiting scheme [68].

In addition to the general concepts behind dose escalation in phase I trials described above, multiple novel phase I trial designs have been introduced and proposed as models for use in radiosensitizer trials in order to ensure timely recruitment and completion of phase I studies. Three examples are briefly described.

### 3.6. Phase 0 Studies

The Phase 0 or window-of-opportunity trial design is viewed as low risk and allows the patients to receive the study drug during the 1 or 2 weeks prior to RT [69,70]. Randomized studies where patients receive study drug *vs.* placebo are relatively straightforward, and useful biomarker data can be generated. The obvious disadvantage of this design is that the drug is not administered together with RT. On the other hand, this may be perfectly suited for clinical trials with immunomodulators such as vaccines or immune checkpoint inhibitors, where there is a paucity of reliable preclinical data, and the activity of the drug may be potentiated by RT even though it is not administered concurrently [71].

### 3.7. Drug Duration Escalation Study

The objective of this study design is to escalate the total number of fractions of RT that are given in conjunction with the systemic agent. A standard drug dose is administered throughout the study, but successive cohorts of patients receive both the drug and RT for progressively longer periods of time [11,64].

### 3.8. Ping-Pong Design

The ping-pong (or flip-flop) design is particularly useful in studies with RT [72]. By way of background, in phase I trials of targeted agents alone, the dose-limiting toxicity is usually apparent within a few days or weeks, generally before the next cycle is due [11]. However, when trials involve RT, it may be appropriate to wait longer—Possibly two to three months—Before escalating or de-escalating the dose of the systemic agent [6]. This typically interrupts continuity of patient accrual. In the ping-pong design, patients can be accrued to the drug B cohort while awaiting maturation of toxicity data from drug A. A current example of this trial design is the “DREAM” study enrolling in the UK. This trial is investigating the addition of cediranib (AZD2171) and a MEK inhibitor (AZD6244) to standard chemoradiation in patients with rectal cancer. Patients are randomized to one arm or the other, and the randomization alternates between cediranib and AZD6244 [73].

### 3.9. Phase II Trial Designs with Radiosensitizers

It is recommended that randomized phase II trials be performed in place of single-arm phase II studies in order to maximize evaluation of true clinical activity [74–76]. This obviates the need to rely on historical control data when deciding if the combination therapy is superior to RT alone. Primary endpoints are based on tumor response or other encouraging surrogate endpoints, which allows molecularly targeted agents to move forward more quickly to phase III trials. Other useful designs include studies investigating multiple agents compared to a standard therapy control group. This allows pilot efficacy testing of each targeted agent against the control, although these studies require statistical adjustment for multiple comparisons. Additional recommendations regarding phase II trial design can be found in the NCI-RTOG Translational Program Strategic Guidelines published by Lawrence *et al.* [6].

### 3.10. Trial Funding and Development

It is estimated that two thirds of all cancer patients receive radiation therapy along the course of their disease. In general, development of and support for combination RT trials is significantly less compared to non-RT trials. The reasons for this fact are multifactorial and beyond the scope of this review; however, competition will likely remain fierce for government funding in the coming years, and one potential avenue for research funding and trial sponsorship that is worth consideration is the pharmaceutical industry. In a recent review, Ataman *et al*. from AstraZeneca conclude that despite the challenges, radiation therapy combination studies with molecularly targeted agents represent a significant opportunity in their view. However, they found that trials proposing combinations of novel agents with RT were more likely to be supported by individual cancer institutes or cooperative groups as opposed to the pharmaceutical industry. Further, they found that most phase I/II RT combination trials occur following approval of the novel agent as a monotherapy or in combination with chemotherapy. Thus, novel agents that fail in single- or combination chemotherapy trials will often not be tested as radiosensitizers [13]. Although targeted radiosensitizers are often cytostatic and many may have limited benefit as monotherapies, they could have substantial benefit in combination with RT [6]. Thus, the *opportunistic* pattern of trial development for novel molecularly targeted agents in combination with RT prohibits systematic investigation of their role as radiosensitizers.

This *opportunistic* pattern further suggests that radiosensitization may not be viewed as an independently fruitful basis for drug and trial development [13]. Lawrence *et al.* identify possible barriers that further contribute to lack of enthusiasm among pharmaceutical companies: (1) Trial designers may be concerned about excess toxicity from combining novel agents with RT; if such toxicity data comes to the attention of the public and regulatory bodies early in the development of the agent, this may lead to delayed approval or irreparably mar the drug’s reputation; (2) Trial development with RT involves a number of non-biological factors such as treatment volume definitions and quality assurance procedures that must be standardized and overseen in a manner not typically required in the administration of systemic agents; (3) Trials combining RT with novel agents tend to arise approximately eight years into the lifetime of the drug; because of reduction in the remaining time under patent, such trials may become less appealing for pharmaceutical companies to support [6].

Proposals to develop multi-study agreements between pharmaceutical companies and interested academic institutions to overcome these barriers are warranted [13]. Standard funding algorithms to support academia-initiated studies would be a reasonable solution to the relative lack of funding in trials that incorporate RT and targeted agents. Incorporating pre-clinical work into such agreements creates a logical framework that allows a smooth and rational transition between pre-clinical and clinical studies. An attempt to better develop communication and links between academia and industry would be to have exchange programs where radiation oncologists and industry personnel to spend time in the others’ work environment to improve communication and foster idea exchange [13]. Such a model has already been undertaken at AstraZeneca.

## 4. Prostate Cancer: Overview and Rationale for Targeted Radiosensitizer Development

RT is commonly used in the management of prostate cancer, which is the most frequently diagnosed cancer among American men and accounts for an estimated 28,170 deaths per year [77]. Standard of care treatment paradigms for early-stage prostate cancer include active surveillance, surgery, external beam RT, and brachytherapy, while more advanced localized disease is generally managed with a combination of modalities and frequently includes the addition of androgen deprivation therapies (ADT). The mainstay of metastatic disease management is ADT, with chemotherapy, immunotherapy, and additional experimental options considered for castration-resistant disease [78].

While early stage prostate cancer managed with RT is associated with a high rate of biochemical control, greater than 30% of patients with more advanced localized disease can experience biochemical failure following management with RT [79–81]. Current strategies employed to improve outcomes include the addition of ADT and radiation dose escalation. Both strategies have successfully reduced biochemical failure rates: 3–6 months of neoadjuvant and concurrent ADT added to RT improves biochemical control by approximately 15%–25% across risk categories [79,82–86], whereas dose escalation to 78 to 80 Gy improves biochemical control by approximately 10%–20% [86–89]. However even with these improvements, biochemical failure rates remain poor for patients with higher risk localized disease and locally advanced disease; for example, after 4 months of neoadjuvant and concurrent ADT, 10-year biochemical failure rates were 28% and 31% for intermediate- and high-risk localized groups, respectively, in the recently published data from RTOG 94-08 [79]. Meaningful biochemical control rates are even lower for locally advanced patients. Moreover, there has been no consistent demonstrable improvement to overall survival with dose escalation in randomized trials, and despite survival benefits with the addition of ADT to RT, overall survival for advanced prostate cancer leaves substantial space for improvement. The 10-year overall survival for patients on RTOG 94-08 with intermediate risk disease was 61% following 4 months of neoadjuvant and concurrent ADT; with the addition of 2–3 years of adjuvant ADT for high-risk and locally advanced patients, 10-year overall survival remained 54% to 58% [80,90]. Additionally, prolonged courses of ADT are associated with considerable toxicities and further dose escalation strategies are limited by normal tissue toxicity thresholds [91].

Thus, these data support the need for novel strategies to improve control of locally advanced prostate cancer, including the development of novel molecularly targeted radiosensitizers. There are clear advantages to studying radiosensitizers in this patient population, including ample study participants due to its relatively high incidence as well as the ability to use intermediate endpoints including biochemical progression free survival and pathologic response rates with post-treatment biopsies to allow for more timely evaluation of trial outcomes. Moreover, physical radiation dose escalation and beam conformality has approached an upper limit with prostate cancer external beam RT. Tumor-targeted radiosensitizers may offer a means for further biological dose escalation with relative sparing of normal tissue [92,93].

## 5. Candidate Targeted Agents as Radiosensitizers in Prostate Cancer

In the remainder of this manuscript, we will review a select number of promising novel molecularly targeted agents that may serve as radiosensitizers amenable to clinical study for high-risk localized and locally advanced prostate cancer. Table 1 further reviews recent trials investigating targeted agents used neoadjuvantly, concurrently or adjuvantly with RT for prostate cancer.

### 5.1. Heat Shock Protein 90 (HSP90) Inhibitors

The non-oncogene addiction or stress response machinery presents an intriguing option for cancer therapeutics. Existing in highly proteotoxic environments, tumor cells are subjected to chronic and acute hypoxia, increased levels of DNA damage, high levels of reactive oxygen species, and protein complex imbalances due to aneuploidy [94]. Survival under these conditions is enabled by the aid of efficient cellular stress response machinery, such as heat shock proteins (HSP). In mammals, the heat shock protein family is categorized into 4 major subgroups based on their molecular weight: HSP90, HSP70, HSP60 and small HSPs (15–30 kDa). The higher molecular weight HSPs are ATP-dependent proteins, while the smaller molecular weight HSPs function in an ATP-independent manner [95]. The survival advantage afforded by HSPs to cancer cells is via stabilization of misfolded proteins preventing protein aggregation and association with key proteins involved in both apoptotic-dependent and -independent cell death and cell survival pathways. Under stress conditions, they are also responsible for selective stability and degradation of client proteins [96].

HSP90, a member of this family, is a ubiquitous molecular chaperone overexpressed in a variety of cancers, including prostate cancer [97]. The HSP90 structure essentially consists of three domains: (a) an *N*-terminal domain responsible for its ATPase activity required in client protein folding; (b) a charged bridging region with affinity for co-chaperones and client proteins; and (c) the *C*-terminal domain that contains a tetratricopeptide repeat-binding (TPR) motif which recruits similar repeat containing co-chaperones such as HOP (HSP organizing protein), is involved in dimerization, and regulates its ATP dependent activity [98].

HSP90 can be further subdivided into HSP90α, HSP90β (or glucose related protein 94 (GRP94)) and tumor necrosis factor receptor-associated protein 1 (TRAP1). While the expression of HSP90α is inducible and tissue specific, HSP90β is constant and ubiquitous [99–102]. HSP90α and HSP90β share 86% homology and are predominantly present in the cytoplasm, though about 10% of the total resides in the nucleus. HSP90α can also be found at the cell surface and in the extracellular space.

HSP90 inhibition offers a multi-pronged attack on many aberrant pathways critical for prostate tumor maintenance and intrinsic radioresistance given the diverse clientele of HSP90 [103,104]. HSP90 client proteins include transcription factors, cell cycle regulators, signaling kinases, mediators of apoptosis and steroid hormone receptors, including the AR. AR is critical for prostate cancer growth and survival [105]. HSP90 is required for the stabilization of active conformations of AR and also binds and stabilizes AR in inactive conformations. HSP90 is also involved in the nuclear translocation of cytosolic AR, and this nuclear transport is necessary for binding to the androgen response elements (ARE) in promoter regions of DNA, facilitating subsequent AR-dependent transcriptional programs [106]. Apart from AR-dependent pathways, those responsible for cell cycle arrest, DNA damage response and repair and those attributed to radioprotection such as the PI3K-Akt-mTOR pathway, have protein components that are stabilized by HSP90 [104,107–111].

The PI3K-AKT-mTOR and AR pathways appear to play a particularly important role in counter-regulating each other in prostate cancers, such that inhibition of both pathways has recently been shown to result in synergistic cell killing in preclinical models [103]. One proposed consequence of androgen suppression is upregulation of the PI3K-AKT-mTOR pathway leading to increased radioresistance. Inhibition of multiple components of the PI3K-AKT-mTOR axis in cancer cells is particularly attractive as mTOR exerts negative feedback regulation on AKT1 as demonstrated by the limited efficacy of rapamycin or its analogs. Thus, pharmacological blockade of HSP90 can overcome signaling redundancies and mechanisms of drug resistance commonly observed in many cancers and simultaneously targets two major tumor maintenance pathways in prostate cancer cells, the PI3K-AKT-mTOR and AR pathways.

The first class of HSP90 inhibitors to be examined were the geldanamycin analogues (binding to the ATP pocket of HSP90), specifically 17-allylamino-17-demethoxygeldanamycin (17-AAG) and 17-(dimethylaminoethyl-amino)-17-demethoxygeldanamycin (17-DMAG), which have been exhaustively characterized preclinically and have also been tested in several phase I and II clinical trials [116–119]. Previous studies with geldanamycin derivatives have been valuable as a proof of concept, showing that inhibition of HSP90 has anti-cancer and radiosensitizing properties in several tumor-derived cell lines *in vitro* (including prostate, lung, colorectal, glioma, and pancreatic carcinomas) and *in vivo* through tumor xenograft models (human cervical, prostate and head and neck squamous cell carcinoma) [120–126]. While in theory targeting HSP90 seems highly promising, results from clinical trials used as monotherapy have been modest. The factors associated with the limited clinical success of these compounds include poor solubility, difficulty in formulation, inconsistent pharmacokinetics, hepatotoxicity, susceptibility to P-glycoprotein efflux and polymorphic metabolism by NQO1/DT-diaphorase enzymes [127].

The need to overcome these limitations and improve the efficacy of geldanamycin analogues led to the development of next generation HSP90 inhibitors that do not possess many of these limitations. NVP-AUY922 (AUY922), a resorcinol isoxazole, is one of the most potent synthetic small molecule inhibitors of HSP90 [120]. Single agent AUY922 has shown potent preclinical anti-cancer activity *in vitro* and *in vivo* against a range of histologic cell types including head and neck squamous cell carcinomas (HNSCC), pancreas, prostate, lung, cervical, colorectal, breast carcinomas, myelomas, melanomas, sarcoma and glioblastoma [120,128–131]. Ganetespib is a unique triazolone-containing small molecule inhibitor of HSP90, structurally unrelated to the ansamycin class, which also exhibits potent activity in a broad range of preclinical models of human malignancies [132]. Moreover, ganetespib displays superior pharmacological and safety properties compared to other next generation HSP90 inhibitors.

The *in vivo* efficacy of HSP90 inhibition for radiosensitization has been studied in a limited fashion. Of the four studies reported, three involved the geldanamycin derivative 17-AAG used in prostate, cervical, and HNSCC human tumor xenograft models, while only two studies have tested AUY922 (using HNSCC and prostate xenograft models) [93,110,123,133]. HSP90 inhibition offers the theoretical possibility of potent radiosensitization through broad downregulation of multiple critical radioresistance pathways whose components are members of the HSP90 clientele, such as signal transduction pathways (PI3K-AKT-mTOR) [104,107,108] and DNA damage response (DDR) pathways (ATR/Chk1) [123,134,135]. Our study with AUY922 and prostate cancer cells suggests that it may indeed impart radiosensitization through multiple mechanisms: (1) reassortment of prostate cancer cells into G2-M; (2) downregulation of the PI3K-AKT-mTOR radioresistance pathway; and (3) downregulation of the ATR-Chk1 DDR pathway. Other recent studies have shown that the ATR-Chk1 DDR axis is also a client pathway of HSP90 in HeLa and MCF7 cells [134]. These findings support the results of our γ-H2AX foci assay in which we observed increased production and persistence of radiation-induced DNA DSBs in cells treated with AUY922. Our observations that AUY922 causes a G2-M cell cycle arrest and downregulates components of the PI3K-AKT-mTOR pathway were consistent with other studies in non-prostate cancer cell lines [136,137]. With such compelling pre-clinical data we believe that HSP90 inhibition serves as a promising adjunct to RT and that combination treatment is warranted. A clinical trial combining ganetespib and external-beam RT for men with localized prostate cancer is in development by our group.

### 5.2. Sunitinib and Sorafenib

Sunitinib malate is a potent oral inhibitor of multiple tyrosine kinase receptors with preclinical assays demonstrating activity against the vascular-endothelial growth factor receptor (VEGFR2), platelet-derived growth factor receptor (PDGFRα and PDGFRβ), C-Kit, and Fms-like tyrosine kinase-3 (FLT3) [138–140]. Early reports suggested that sunitinib’s interaction with the VEGFR and PDGFR pathways may enhance the cytotoxic effects of radiation. *In vitro* studies with pancreatic cancer cell lines treated with combined sunitinib and radiation showed reduced clonogenic survival, while *in vivo* assays showed delayed tumor growth [141]. Similar *in vivo* results were seen in a Lewis carcinoma mouse model, which also demonstrated radiation-induced endothelial cytotoxicity, resulting in tumor vascular destruction [142]. Both models linked sunitinib’s effect to the attenuation of signal transduction through radiation-induced survival pathways, in particular PI3K-AKT and MAPK-ERK pathways.

Preclinical and clinical studies have also explored sunitinib as a radiosensitizer for prostate cancer. Brooks *et al.* examined sunitinib in three prostate cancer cell lines [143]. Two of these cell lines were hormone-independent (DU145 and PC3) and expressed PDGFR and/or VEGFR, while the third was an androgen-dependent cell line (LNCaP), which did not express these targets. As expected, sunitinib reduced clonogenic survival in DU145 and PC3. Of note, sunitinib suppressed radiation-induced phosphorylation of ERK, providing more evidence that sunitinib’s effect may be mediated through blockade of this pathway. Interestingly, in that xenograft model, sunitinib did not delay tumor growth when administered concurrently with radiation but did enhance growth delay when administered one day after radiation treatments had been completed. The authors hypothesized that the positive outcome seen with delayed sunitinib suggests that the drug may suppress the ability of irradiated tumor stroma to sustain re-growth. Indeed, reports have demonstrated improved tumor control when anti-angiogenic agents are given after completion of RT [144]. Alternately, the differential effect of delayed *versus* concurrent sunitinib may be explained by the creation of a hypoxic environment that undermines potential radiosensitization, an effect seen in other anti-angiogenic agents [145]. Reports suggesting improvement in tumor blood flow and reduction of hypoxia with angiogenesis inhibitors are also present [146,147]. Certainly, additional work is needed to better elucidate the mechanisms by which sunitinib interacts with RT when administered both during and after treatment.

Regardless of the preclinical data, sunitinib has been moved into clinical trials as a radiosensitizer for prostate cancer. A multi-institutional phase I study has defined the maximum tolerated dose of 20 mg/day of sunitinib when administered neoadjuvantly, concurrently, and adjuvantly with standard RT and androgen deprivation therapy in high-risk, localized prostate cancer patients [112]. Other active clinical trials include a phase II study examining the combination of sunitinib with docetaxel prior to salvage RT in post-prostatectomy patients experiencing a biochemical recurrence [148]. This study design is predicated on positive results seen with the combination of sunitinib and docetaxel in patients with metastatic castrate-resistant prostate cancer, and the demonstrated safety of this combination [149]. Additionally, a phase 1 study is examining sunitinib when administered prior to prostatectomy in high-risk, localized patients undergoing prostatectomy, and should provide valuable histologic information [150].

Closely related to sunitinib is sorafenib, which is also a small molecule tyrosine kinase inhibitor of both VEGFR and PDGFR. Sorafenib also carries activity against Raf kinases. Similar to sunitinib, early preclinical and clinical studies have shown the potential for enhanced effect when combined with radiation [151,152]. Sorafenib has also been moved into a combined phase I/II trial exploring its use in combination with standard RT and hormonal therapy for high-risk, localized prostate cancer patients [153].

### 5.3. Dasatanib and Other SRC Inhibitors

A large body of evidence has implicated SRC, a non-receptor tyrosine kinase, as an important target in prostate cancer. SRC is highly expressed in prostate cancer cell lines, and *in vitro* studies have shown decreased proliferation, invasion, and migration after exposure to SRC inhibitors [154–156]. *In vivo* studies have also demonstrated reduced prostate cancer growth with SRC inhibitors [157]. Exploration of the mechanisms of SRC signaling has shown this pathway to be important for both androgen-dependent proliferation as well as androgen-independent growth [158].

Dasatinib is an oral tyrosine kinase inhibitor that targets the SRC family kinases in addition to BCR-ABL, C-Kit, and PDGFR. Preclinical study of dasatinib with PC-3, DU-145, and LNCaP has demonstrated efficacy in its ability to impair migration and invasion [159]. In the same report, dasatinib significantly undermined the ability of PC-3 cells to induce angiogenesis *in vivo*. Key downstream pathways that were affected by dasatinib included AKT, FAK, and STAT3. Given that dasatinib is involved in important aspects of the metastatic cascade, a phase II trial was initiated to test dasatinib as monotherapy in chemotherapy-naïve patients with metastatic castrate resistant prostate cancer (mCRPC) [160]. Results from this trial were modest, which prompted combination of dasatinib with docetaxel in a phase I/II trial in the same population [161]. This trial showed a much better objective response rates with 30% of patients having radiographic resolution of osseous metastases on bone scan. Unfortunately, a large randomized phase III trial comparing docetaxel versus docetaxel plus dasatinib for men with metastatic CRPC failed to show clinical improvements using the combination therapy [161].

Studies examining SRC inhibitors as radiosensitizers are more limited. Cuneo *et al.* studied the effect of the SRC inhibitor SU6656 on human umbilical endothelial cells and found decreased clonogenic survival when combined with radiation along with decreased capillary tubules, which served as a marker for angiogenesis [141]. This group also studied SU6656 in a Lewis carcinoma model, which demonstrated enhanced tumor growth delay and increased radiation-induced destruction of blood vessels. Similar enhanced sensitivity to radiation was seen with other SRC inhibitors in lung cancer and head and neck squamous cell carcinoma cell lines, with dasatinib being used in the latter [162,163]. Given these preclinical results, attempts have been made to move dasatinib into clinical trials. Unfortunately, dasatinib was not able to be safely combined with chemoradiation incorporating carboplatin and paclitaxel for stage III non-small cell lung cancer due to increased rates of pneumonitis [164]. However, a phase I trial examining dasatinib in combination with androgen deprivation therapy and RT for intermediate- and high-risk, localized prostate cancer patients is now ongoing [165].

### 5.4. mTOR Pathway Inhibitors

As discussed above, the PI3K-AKT-mTOR pathway has been implicated as a radiation-survival pathway across multiple cancers. PTEN is a suppressor of the PI3K-AKT pathway, and genetic alterations/deletions in PTEN are commonly seen in prostate cancer, with some estimates suggesting decreased PTEN expression in over 80% of primary prostate cancers [166]. Dysregulation of PTEN is also associated with particularly aggressive prostate cancer phenotypes [167].

Mammalian target of rapamycin (mTOR) is a downstream kinase of AKT. Given the high prevalence of PTEN loss/inactivation and the importance of this pathway to tumor behavior and response to radiation, inhibition of mTOR activity has been an attractive therapeutic target in prostate cancer. *In vitro* assays with everolimus, an mTOR inhibitor, showed a significant decrement in clonogenic survival with the addition of everolimus to RT, particularly in those cells that were PTEN deficient [168]. Enhanced radiosensitization with everolimus was seen in both androgen-sensitive and androgen-independent cell lines [169]. Interestingly, administration of everolimus after completion of radiation led to the strongest cytotoxic effect *in vitro*, which may be a result of cell cycle kinetics, as mTOR inhibitors tend to shift a larger proportion of cells into the radioresistant G1 phase. Clinical exploration of everolimus is ongoing with two active phase I trials of everolimus in combination with standard RT and hormonal therapy for high-risk, localized and locally advanced prostate cancer [170,171]. Additionally, a phase 1 trial is also examining the combination of everolimus and salvage RT for post-prostatectomy patients experiencing biochemical recurrence [172]. Other agents targeting mTOR in prostate cancer include the dual PI3K-mTOR inhibitor, BEZ235, which has shown potent *in vitro* radiosensitization both in normoxic and hypoxic conditions [173]. While BEZ235 is being examined clinically in the mCRPC population, it is yet to be combined with radiation in a clinical trial.

### 5.5. Androgen Deprivation Therapy

Although out of the intended scope of this article, we would be remiss if we did not briefly review the role of ADT combined with RT. As previously discussed, neoadjuvant and concurrent ADT has generally been shown to improve biochemical progression free survival and overall survival rates in locally advanced prostate cancer [79,82–85], and the addition of adjuvant ADT further improves these outcomes in high-risk prostate cancer [80,90]. Mechanisms by which ADT and RT may interact are not well-established, although a number of *in vitro* and *in vivo* studies have sought to answer the question.

Relevant *in vivo* animal models include the Shionogi *in vivo* tumor system and the R3327-G Dunning rat prostate tumor model. In the Shionogi *in vivo* tumor system, a spontaneous murine mammary carcinoma that was found to be androgen dependent was grown as allograft in mice with severe combined immune-deficiency. Investigators then performed orchiectomies for androgen deprivation, either neoadjuvantly at 12 days prior to RT or adjuvant at 1 to 12 days following RT. Radiation response rate, as defined as the dose required to control 50% of tumors (TCD_50_), was significantly lower if androgen deprivation was administered prior to RT, reflecting the importance of sequencing of ADT relative to RT. The authors surmised that the mechanism of reduction in TCD_50_ with neoadjuvant ADT may be due to cytoreduction leading to the need to eradicate fewer clonogens, volume reduction decreasing a hypoxic fraction, or via synergistic interactions through apoptotic pathways [174].

In a second *in vivo* model, R3327-G Dunning rat prostate tumor models were grown the flanks of rats. Again, androgen deprivation was achieved with orchiectomy, and a total of seven experimental groups and controls were subjected to various sequencing of RT, orchiectomy and androgen restoration with testosterone implants. Results showed that mean tumor doubling time was most suppressed for the group receiving neoadjuvant ADT, even when compared with groups receiving concurrent or adjuvant ADT. The authors concluded that in addition to potential increased overall cell killing, ADT may decrease growth velocity of surviving cancer cells when combined with RT [175,176].

Whether ADT is acting as a radiosensitizer *per se* cannot easily be tested *in vivo*, and as such, *in vitro* analyses have sought to answer this question. One such study exposed groups of LNCaP cells to neoadjuvant androgen deprivation and RT and found that there was a consistent supra-additive increase in apoptosis for cells exposed to the combination regimen as opposed to either ADT or RT alone. However, overall cell death as determined by clonogenic survival did not support significant radiosensitization by ADT despite this supra-additive apoptosis [177]. Another *in vitro* study investigated the effect on incubating LNCaP and PC-3 cells in goserelin followed by RT. Similarly, this study found no significant effects of goserelin incubation on clonogenic survival or cell viability as compared to RT alone [178]. These studies concluded that the *in vivo* observation of increased tumor control from the combination of ADT and RT cannot be attributed to increased ADT-induced radiosensitivity.

A number of additional explanations for the ADT-RT interaction have been investigated, including the possible role of ADT in potentiating male host immunity by androgen deprivation [179], which may increase immune-mediated tumor kill.

Despite lack of clarity of the mechanism of interaction, combining RT with agents targeting the androgen receptor signaling axis offers an appealing basis for future trial design. Tsao *et al.* [180] provide an excellent review of novel agents targeting this axis including abiraterone and MDV3100.

## 6. Conclusions and Future Directions

Although novel targeted molecular radiosensitizers have the potential to significantly improve cancer outcomes, there has been relatively limited preclinical and clinical investigation of such promising agents. As we have nearly exhausted our ability to improve radiation conformality and dose escalation, it is critical that we pursue biologic methods of exploiting inherent weaknesses within the tumor using molecularly targeted agents in an effort to increase the therapeutic ratio of radiation. There is gaining momentum in support of using targeted agents as radiosensitizers, and guidelines for preclinical and clinical studies have been published by various groups including the NCI and RTOG. We have reviewed the challenges and summarized the various recommendations for preclinical efforts and clinical trial design with targeted radiosensitizers. These efforts towards incorporating molecularly targeted agents with RT are worthwhile but in order to be successful will require better patient stratification using robust biomarkers incorporated into novel clinical trial designs. Additionally, we have detailed the rationale and evidence supporting the application of select molecularly targeted radiosensitizers such as HSP90 inhibitors, tyrosine kinase inhibitors, and mTOR inhibitors in the treatment of prostate cancer.

## Figures and Tables

**Table 1:** Recent trials investigating targeted agents used neoadjuvantly, concurrently or adjuvantly with radiotherapy for prostate cancer *.

Radiosensitizer	Risk group	Target	Trial number **	Trial phase	Trial status	Outcomes
Semaxanib + ADT	Intermediate-to high-risk	VEGF receptor	NCT00026377	I	Completed	See note †
Sunitinib + ADT	High-risk	Multi-targeted RTK	NCT00631527	I	Completed	Feasibility achieved with recommended phase 2 dose of sunitnib (25 mg daily) [112]
Panobinostat	High-risk	HDAC	NCT00670553	I	Completed	-
Everolimus + ADT	High-risk	mTOR	NCT00943956	I	Unknown ‡	-
Everolimus	Biochemical recurrence (salvage)	mTOR	NCT01548807	I	Recruiting	-
Everolimus + ADT	High-risk	mTOR	NCT01642732	I	Recruiting	-
Dasatinib + ADT	Intermediate-to high-risk	SRC	NCT01826838	I	Recruiting	-
Ganetespib + ADT	High-risk	HSP90	Pending	I	Pending	-
Sorafenib + ADT	Intermediate-to high-risk	Multi-targeted RTK	NCT00924807	I/II	Terminated	-
Bevacizumab + ADT	High-risk	VEGF receptor	NCT00349557	II	Completed	Bevacizumab + ADT does not exacerbate acute side effects but may worsen late effects following IMRT [113]
Sunitinib + docetaxel	Biochemical recurrence (salvage)	Multi-targeted RTK	NCT00734851	II	Active but not recruiting	-
TAK-700 + ADT	High-risk	CYP17A1	NCT01546987 (RTOG 1115)	III	Recruiting	-

* Adapted from Palacios, *et al.* [114];

** As listed on USA National Institutes of Health’s ClinicalTrials.gov registry; ADT—Androgen deprivation therapy; VEGF—Vascular growth factor; RTk—Receptor tyrosine kinase; HDAC—Histone deacetylase; mTOR—Mammalian target of rapamycin; HSP90—Heat shock protein 90; CYP17A1—Cytochrome P450 17A1;

† A phase II trial of SU5416 by the same author investigating its use in hormone-refractory prostate cancer states that additional study of SU5416 in prostate cancer patients is not recommended given negative results of the phase II trial [115];

‡ The recruitment status of this study is unknown because the information has not been verified recently on clinicaltrials.gov.
